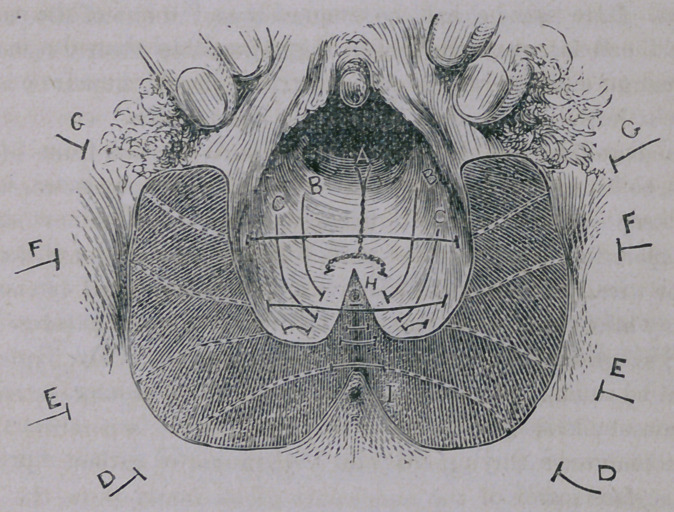# Operation for Complete Laceration of Perinæum

**Published:** 1870-09

**Authors:** C. C. F. Gay

**Affiliations:** Surgeon to the Buffalo General Hospital


ART. III.—*Operation for Complete Laceration of Perinæum*. By C. C. F. GAY, M. D., *Surgeon to the Buffalo General Hospital*.

Laceration of the perineum is complete when the recto-vaginal septum to a greater or less extent is destroyed. It is complete when the laceration is so great as to throw the rectal outlet and the vaginal inlet into one cavity. I have endeavored to represent in process of repair the appearance of the parts after this injury in the accompanying diagram. The cut represents the recto-vaginal septum torn through, leaving the anus obliterated.

*I*, represents the normal location of the anus, but now, after the rupture, if the parts are examined by the fore finger, it will be found necessary, in order to find an opening into the rectum, to glide the finger along the rupture upwards, for an inch or two inches, when an opening will be felt, which might be mistaken by the inexperienced for a fistulous opening through the vaginal walls, but this is the outlet now of the rectum. Its location I have represented in the diagram by a point made at *H*.

This laceration is a most serious injury of parturition, and while it cannot always be avoided by the most careful accoucheur, it may always be mended by the skill of the surgeon.

Before the operation, the bowels should be well evacuated by a dose of castor oil.

The patient, anaesthetized, lies upon the back, in position for operation for lithotomy. An assistant upon each side the patient supports the limbs with one hand, and with the fingers of the other hand keeps open the labia.

The end of the left fore finger of the operator, with its palmar aspect upwards towards the uterus, is thrust into the opening of the rectum, for the purpose of holding the parts to be denuded of mucous membrane tense. Sharp pointed scissors makes the best instrument to use for denuding the bowel portion, and no difficulty will be experienced in readily preparing a raw surface upon either side the fissure, and which should rise up to a point a half inch or more above the point of opening into the bowel, with three eighths or one half inch of denuded surface on either side.

This accomplished, the next step in the operation is to denude the labial portion; for this purpose either the scissors or knife may be used. The scar of the lacerated surface will serve as guide somewhat in scarifying; and in scarifying it is better to begin below, so as not to be embarrassed by the dripping of blood from above. More tissue should be removed than the line of the cicatrix would call for, or extended over a greater labial than the original perineum, and the width of raw surface should measure one half inch at least. A greater width would be desirable.

In denuding the opposite side, the parts should, from time to time, be brought together, to see that both are equally pared, so that the coaptation of scarified surface be perfect. The next step in the operation is to bring the parts together and secure them with silver wire. The diagram will give a better idea than I can convey in any other way, of the manner of introducing the sutures. No quill sutures, as recommended by Baker Brown, or Dr. Gross, are required; neither is it necessary to divide the sphincter, as recommended by these gentlemen.

In the accompanying diagram the granulated surface represents the surface scarified.

*A*, the first suture, passed and twisted near the mouth of the uterus; *B* and *C* sutures passed, but not twisted. *D* the first perineal suture passed along the edge of the anus.

*E*, near the same, or first suture; *G* and *F* cross from one side of the vagina to the other.

The dotted lines show the continuation of the sutures within the tissues. The sutures should not be twisted tightly, but only sufficiently tight to coaptate the parts; subsequent swelling of the parts will tighten the sutures; they should, therefore, be but loosely twisted, or, more properly, I should say, the surface should be loosely brought together. The vaginal sutures, after twisting, should be turned upwards, in the direction of the axis of the vagina, left about one inch long, and the end mashed, so that they may not injure the surface of the vaginal walls. The perineal sutures are left about the same length.

The latter may be removed about the eighth day after the operation ; the former should remain eighteen or twenty days, and can best be removed by the aid of vaginal speculum. After the operation, the knees are held loosely together by a bandage, cold water dressings applied to the wound, and the bowels constipated by just sufficient opium to accomplish this purpose, and should not be allowed to move for eight or ten days. The use of the rectal tube is quite important, as it enables the flatus to escape without passing between the wounded surfaces, and thus tending to prevent union. The urine should be drawn off by the catheter.

## Figures and Tables

**Figure f1:**